# Pilot Trial of Workable: A Therapist-Supported Digital Program for Injured Workers

**DOI:** 10.3390/ijerph20032460

**Published:** 2023-01-30

**Authors:** Joanna Crawford, Jay Spence, Tali Lovegrove, Edman Tam, Daniel Collins, Samuel B. Harvey, Mark Deady

**Affiliations:** 1 Black Dog Institute, Faculty of Medicine, University of New South Wales, Sydney, NSW 2031, Australia; 2 Uprise Services Pty Ltd., Sydney, NSW 2000, Australia

**Keywords:** sick leave, return to work, eHealth, online intervention, blended intervention, satisfaction, depression, anxiety, absenteeism, mental health

## Abstract

Workplace sickness absence is a major public health and economic problem, and common mental disorders (CMDs) such as anxiety and depression are associated with particularly high rates of long-term sickness absence. Effective return-to-work (RTW) interventions are required. This pilot study investigates the feasibility, acceptability, and potential effectiveness of a new therapist-assisted Web-based RTW intervention (*Workable)* for injured workers on sick leave for a psychological or physical injury. A single-group open pilot trial design was used, with assessments at pre-treatment and post-treatment. The intervention consisted of 6 weeks of online modules and 6 coaching calls from a psychologist. A total of 13 participants were recruited and 9 completed all questionnaires. Program adherence was high, with 92% of participants completing the 6-week intervention. Participants reported high levels of intervention satisfaction and ease of use. There were large and significant reductions between pre- and post-treatment on measures of depression, anxiety, stress, and workdays missed over the past four weeks, along with a significant increase in self-reported work ability. These results suggest that *Workable* is a feasible and acceptable intervention for injured workers, with the potential to improve mental health and RTW outcomes. A randomized controlled trial is required to determine the efficacy of the intervention.

## 1. Introduction

Workplace sickness absence is a major public health and economic problem. Long-term sickness absence from work affects society through loss of productivity and work disability claims [1]. Further, extended sickness absence has a variety of negative impacts on workers themselves, including social isolation, loss of income, and reduction of meaningful activity [2]. Sickness absence is a predictor of future poor health, physical complaints, low mental well-being, and reduced work ability [3]. Longer absences from work are associated with a reduced probability of eventual return to work and subsequent economic and social deprivation [4]. Hence, effective interventions are required to facilitate workers to be able to return to work after becoming unwell.

Mental illness is a leading cause of sickness absence in developed countries [5,6], and is now the most common cause of long-term sickness absence among Australian workers [7]. Anxiety and depression, collectively termed common mental disorders (CMDs) [8], are significantly associated with long term sickness absence from work [9]. Further, people on sick leave for a physical injury or illness, such as musculoskeletal pain, whiplash injury, or cancer, can also experience symptoms of CMD [10,11,12], with emotional distress, fear of returning to work, and cognitive appraisals of pain found to predict longer absence from work in people with back pain [10,13]. A recent meta-analysis of prospective studies found that even amongst people on sick leave for a physical illness, depressive symptoms significantly reduce the likelihood of returning to work [14].

Several reviews and meta-analyses have been conducted examining the evidence for interventions, including psychological therapy, in improving RTW outcomes for workers on sick leave with CMD. A meta-analysis of 16 randomized controlled trials (RCTs) examining RTW interventions involving elements of cognitive-behavioral therapy (CBT) found that although there was a small effect of the intervention group in reducing the number of sick-leave days, the available interventions did not lead to improved RTW rates compared to control groups [15]. A later meta-analysis of 32 RCTs of RTW interventions for people with CMD as well as stress-related, somatoform, or personality disorders concluded that therapy alone did not appear to have an impact on RTW outcomes [16]. However, it did find evidence for interventions involving contact with the workplace, graded RTW, or multiple components (which could include therapy) in improving RTW outcomes. More recently, a 2020 meta-analysis found low-certainty evidence for psychological interventions in reducing the number of sickness absence days compared to usual care, based on 9 studies examining RTW in people with depression [17]. Further, three studies examining antidepressant medication in people with depression yielded inconsistent results for the impact on RTW [17].

Together, these findings suggest that whilst there is a potential role for psychological interventions in improving RTW outcomes in workers on sick leave with CMD, more focused interventions are required, particularly those that focus on work skills or address barriers that may be preventing RTW.

Evidence-based treatments for the improvement of symptoms of CMD, such as CBT and antidepressant medication, do not appear to automatically lead to full occupational functioning. Recent research has found that predictors of RTW for people on sick leave with CMD included positive RTW expectations, high RTW self-efficacy, and high self-reported ability to work [18]. This suggests that interventions aimed at improving RTW outcomes for people with CMD may benefit from specifically targeting expectations and beliefs related to RTW. Indeed, a specific work-focus of psychological interventions for people with CMD has been found to be associated with greater effects on RTW outcomes [19]. For example, a cluster RCT in the Netherlands of a work-focused problem-solving intervention for people returning to work after sickness absence due to CMD found that the intervention was effective in reducing the incidence of recurrent sickness absence and increasing the time to sickness absence compared to care as usual (CAU) [20].

A further future direction to enhance the delivery of RTW interventions is through the use of digital technology. Online delivery has the potential to increase the accessibility of psychological interventions by overcoming barriers such as geographical location, time and travel requirements, and can increase fidelity to evidence-based protocols [21]. Qualitative research has found that digital RTW interventions are viewed as acceptable by people on sick leave with CMD, with a preference for digital RTW interventions that complement traditional RTW support [22]. A recent trial of Web-based CBT for healthcare workers on disability leave found significant improvements in depression and anxiety but did not report RTW outcomes [23].

There have been promising results for both RTW and mental health outcomes using a Web-based intervention, specifically targeting RTW cognitions, developed in the Netherlands [24]. A cluster RCT of the Dutch RTW intervention in 220 sick-listed employees with CMD found a significant effect on the mean duration of first RTW, with the intervention group returning to work, on average, 27 days before participants receiving care as usual (CAU) [24]. In addition, participants in the intervention group had a significantly greater rate of remission from symptoms of CMD compared to those receiving CAU. The Web-based intervention, which was accompanied by support from an occupational therapist, included CBT specifically targeting cognitions regarding RTW, problem solving, and modules on pain and fatigue management [24].

In Australia, a new therapist-assisted Web-based RTW program, *Workable*, has been developed, informed by two Dutch interventions [20,24]. *Workable* is a 6-week online mental resilience program which aims to assist injured workers (i.e., employees on sick leave due to a work-related physical or psychological injury) to return to work earlier. It is based on principles of CBT and problem-solving therapy, specifically targeting RTW. Given that RTW can be affected by symptoms of depression even amongst workers on sick leave for a physical illness or injury [14], *Workable* was designed to be used by employees on sick leave for a physical injury, as well as those on leave for a psychological injury.

The primary aim of this pilot trial was to examine the feasibility and acceptability of the *Workable* program for injured workers. A secondary aim was to conduct a preliminary examination of the potential efficacy of the *Workable* program for mental health (including depression, anxiety, stress, and well-being) and RTW outcomes. This pilot study was the initial step in the evaluation of the *Workable* program. Results of this study can be used to inform any modifications required to the program prior to conducting a larger RCT to examine its efficacy more conclusively.

## 2. Materials and Methods

### 2.1. Design

An open pilot trial design was used to explore the feasibility, acceptability, and effects of the *Workable* program. All participants were allocated to receive the intervention. Participants were assessed using online self-report questionnaires at pre-treatment and 6 weeks later at post-treatment. All participants were invited to complete the post-treatment questionnaires.

The study was approved by the University of New South Wales Human Research Ethics Committee (UNSW HREC) in Sydney, Australia (HC190114) and was prospectively registered with the Australian and New Zealand Clinical Trials Registry (ACTRN 12620000561987).

### 2.2. Eligibility Criteria

Participants were injured workers with a work-related psychological or physical injury in Australia. To be eligible to participate individuals were required to be: (i) aged 18 years or older, (ii) employed, but currently on leave from work due to a work-related physical or psychological injury, (iii) between 2 and 26 weeks into a claim at time of recruitment, and (iv) living in Australia. Individuals were excluded if they: (i) were engaged in legal action with employer, (ii) had difficulty with the English language, (iii) were experiencing severe depression, indicated by a score of ≥20 on the Patient Health Questionnaire-9 (PHQ-9) [25], (iv) were currently experiencing a psychotic episode, or (v) displayed active suicidal intent or a current plan to harm themselves or others.

### 2.3. Sample Size

**Figure 1:**
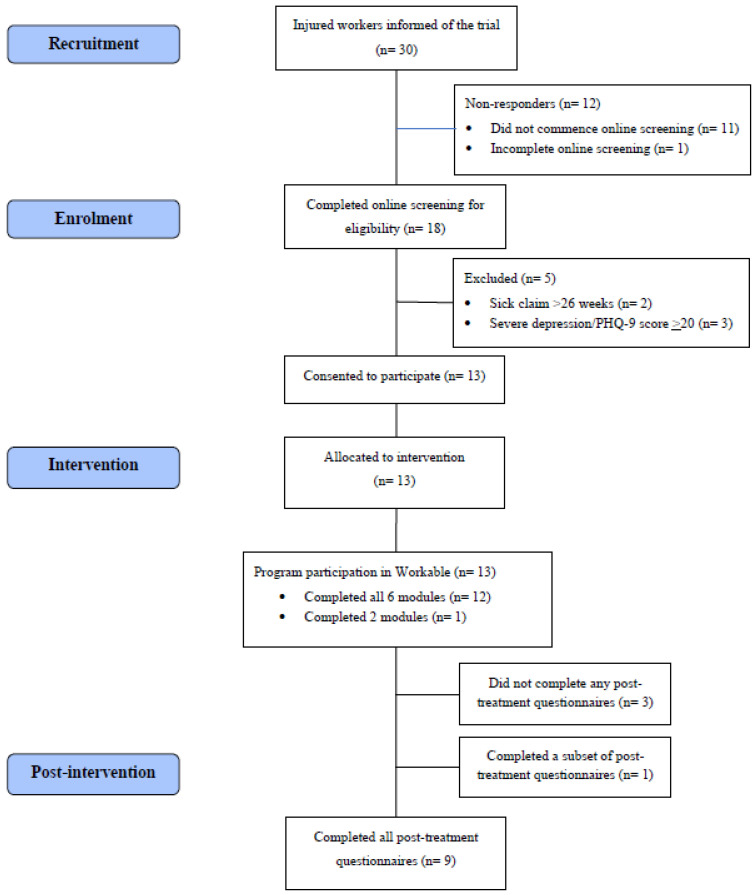
Flowchart of participants.

As this was a pilot study, a formal power calculation was not performed. Previous pilot studies examining the feasibility and acceptability of new digital mental health interventions have included those reporting sample sizes of 9–15 participants [26,27,28,29,30]. A total of 30 injured workers were informed of the *Workable* pilot trial by their injury management specialist or case worker. Of these, 13 (43.3%) met eligibility criteria, provided their consent to participate, and registered for the trial. Figure 1 presents the flow of participants through the trial.

### 2.4. Recruitment and Procedures

Recruitment occurred between the 1st and 23rd February 2021. Participants were recruited through registered service providers with support from Australian state-based insurance provider iCare, including injury management specialists, advisors, and scheme agent case managers. iCare is an insurance provider in New South Wales (NSW), Australia, which provides workers compensation insurance and care services to public and private sector employers and employees in NSW Potential participants were informed of the *Workable* pilot trial by their injury management specialists or case workers (by email or telephone) and applied online at the *Workable* website. Participants were required to complete a brief online screening questionnaire, meet screening criteria, read online participant information, and provide their online consent, prior to registering to participate in the pilot trial. Participants’ first onboarding call with a trial psychologist also functioned as an additional screening process for symptoms of psychosis or suicidal intent.

Participants completed online questionnaires prior to commencing the *Workable* program (“pre-treatment”) and at the end of the 6-week program (“post-treatment”). All participants were emailed an invitation to complete the online post-treatment questionnaires at 6 weeks from baseline, regardless of how many modules of the *Workable* program they chose to complete.

The trial safety protocol specified that any participant who (i) expressed suicidal intent or plan, or (ii) scored below 8/25 on the 5-item WHO Well-Being Index (WHO-5) [31], would be contacted by a psychologist who would undertake a suicide risk assessment and contact appropriate services if required.

### 2.5. Intervention

The Web-based therapist-assisted *Workable* program was developed and delivered in Sydney, Australia by employee assistance provider Uprise Services Pty Ltd. *Workable* is a 6-week online mental resilience program which aims to assist injured workers to return to work earlier. The content of the online *Workable* modules is outlined in Table 1. *Workable* includes weekly core skills modules (of 15 min duration each), including one to four videos per week, and recommended homework exercises (of up to 60 min per week). The content of Workable is based on principles of CBT and problem solving, with specific application to cognitions related to RTW. *Workable* also includes 6 sessions of coaching calls (once a week, for 6 weeks), delivered by a psychologist via teleconference, for a duration of 45 min per call. The coaching calls focused on review of the content of the online modules in *Workable*, and assistance with the implementation of the skills and strategies taught in the online modules. During the coaching sessions, the trial psychologist aimed to focus on work-related examples when discussing skills and strategies with participants. *Workable* was delivered in addition to care as usual (CAU) for participants. However, the trial psychologist did not directly interact with the treating teams of participants.

### 2.6. Measures

#### 2.6.1. Measures to Assess Feasibility and Acceptability

See Table 2 for a schedule of assessment measures. Feasibility was assessed by the recruitment rate, trial attrition (the number of participants who did not complete the pre-treatment and post-treatment measures), and program adherence (the number of *Workable* online modules completed and the number of psychologist coaching calls received).

The Client Satisfaction Questionnaire-8 (CSQ-8) [32] was administered at post-treatment to assess satisfaction with the *Workable* program. The CSQ-8 is a widely used 8-item validated satisfaction measure that has been used across a range of different health services and programs [32,33], including RTW and digital mental health programs [26,34]. This self-administered questionnaire has a high internal consistency (Cronbach’s α = 0.92–0.93) and high concurrent validity in numerous health settings including mental health outpatient settings and substance use populations [32,35]. The CSQ-8 is scored using a Likert scale from 1 (low satisfaction) to 4 (high satisfaction), with total scores ranging from 8 to 32 and higher scores indicating greater satisfaction.

Usability was assessed by a single item, which asked participants to rate on a 5-point Likert scale the extent to which they agreed or disagreed with the statement “I thought that *Workable* was very easy to use.” In addition, qualitative feedback was collected at post-treatment. Participants were given the option to respond to open-ended questions regarding the most and least helpful parts of *Workable*, suggestions for improvements to *Workable* and general comments.

#### 2.6.2. Measures to Assess Potential Effectiveness: Mental Health Outcomes

Symptoms of depression were measured using the Patient Health Questionnaire-9 (PHQ-9) [25]. The PHQ-9 is a self-administered 9-item measure of symptoms of depression over the past two weeks [25,36]. The validity and reliability of the PHQ-9 in primary care and other health settings has been well-established previously [36,37]. The PHQ-9 is dual-purpose and can be used to establish a probable diagnosis of major depressive disorder (MDD) or grade depressive symptom severity with scores ranging from 0–27 [25,38]. Scores of 5, 10, 15, and 20 represent cut-offs for mild, moderate, moderately severe, and severe depression, respectively [36].

Symptoms of anxiety were measured using the General Anxiety Disorder-7 (GAD-7) [39]. The GAD-7, a self-administered sensitive 7-item questionnaire, measures symptoms of generalized anxiety disorder over the past two weeks [39]. Scores range from 0–27 with cut-offs at 5, 10, and 15 to represent mild, moderate, and severe levels of anxiety [39,40]. The GAD-7 is a valid measure for identifying generalized anxiety disorder [40] and is sensitive to assessing symptom severity and detecting change across time, such as change over a course of treatment [39,41].

Perceived stress was measured with the 4-item Perceived Stress Scale (PSS-4) [42]. The PSS-4 is a short version of the widely used PSS [43]. This self-report scale examines, over the past month, the degree to which individuals perceived situations in their life as stressful [42,44]. Scores on the PSS-4 range from 0 to 16, with higher scores indicating higher levels of stress. Population normative data for the PSS-4 have been established [44].

Well-being over the last two weeks was measured using the 5-item WHO Well-Being Index (WHO-5) [31]. The WHO-5 is a widely used, psychometrically sound measure of well-being with high internal consistency, evidence of a one-dimensional factor structure and established validity [45,46]. It consists of 5 positively worded items that are rated on a 6-point Likert scale. Raw scores range from 0 to 25 and are multiplied by 4 to produce a final score, with 0 representing the worst possible quality of life and 100 representing the best possible quality of life [31,45]. Although the WHO-5 was originally developed as a measure of well-being, there is also evidence for its validity in measuring depression [45] and as a depression screening tool [38].

#### 2.6.3. Measures to Assess Potential Effectiveness: RTW Outcomes

Work ability was measured using a subset of three items from the Work Ability Index (WAI) [47]. The WAI is a valid and reliable self-report questionnaire that is used to quantify an individual’s capacity for work and identify employees at high risk of absence, long-term sickness, or disability [48,49]. However, it has been argued that the original WAI, including a list of 51 medical conditions, is a long instrument and can be difficult to fill in [48]. The three items used for this study asked participants to: (i) rate their current work ability overall from 0 to 10 (with 10 indicating ‘able to work at my best’), (ii) rate on a 5-point scale from 1 to 5 their current work ability with respect to the physical demands of the job, and (iii) rate on a 5-point scale from 1 to 5 their current work ability with respect to mental demands of the job. From this, a total score (2 to 20) was generated by summing the three scores, with higher scores indicating a higher perceived work ability. The internal consistency of the three work ability items in this study was moderate (Cronbach’s α = 0.64 to 0.70).

Absence from work was assessed using a single item that asked participants to self-report the number of days (from 0 to 28) in the past four weeks that they had missed a full working day due to their symptoms. Further, at post-treatment, participants were asked whether or not they were back at work, and if so, what type of work (full-time, part-time, casual, contractor, or other) and if it was with the same employer.

### 2.7. Data Analysis

All analyses were conducted in IBM Statistics SPSS 26. Descriptive statistics were used to report sample characteristics and post-treatment indicators of feasibility and acceptability and RTW status. To investigate changes from pre- to post-treatment in mental health outcomes, self-reported work ability, and work absence, the repeated measures linear MIXED model procedure was used for each of the outcome measures. The use of this procedure, with maximum likelihood estimation, allows all available data to be included even when scores for outcome variables at either timepoint are missing. Time was treated as a categorical repeated measures factor. Effect sizes (Hedges *g*, adjusted for sample size) were calculated to determine the size of the within-group change between pre-treatment and post-treatment.

## 3. Results

### 3.1. Baseline Participant Characteristics

Table 3 outlines the baseline characteristics of the sample, including demographics. Participants (N = 13) were adults with a mean age of 47.54 years (SD = 13.43, range = 23–74). Participants were employed in 11 different industry classifications and had been on a claim for a duration of between 2 and 26 weeks at the time of recruitment. The majority of participants (69.2%) reported that they were on sick leave due to a psychological injury (i.e., a work-related mental health issue). However, the remaining 30.8% of participants reported that they were on sick leave due to a physical injury. The mean score on the PHQ-9 at pre-treatment was 14.00 (SD = 4.16, range = 7–19), which is in the moderate range for symptoms of depression. The mean score on the GAD-7 was 12.15 (SD = 3.91, range = 8–21), which is in the moderate range for symptoms of anxiety.

### 3.2. Feasibility

#### 3.2.1. Recruitment Rate

Of the 30 injured workers informed of the pilot trial of *Workable*, 18 (60.0%) applied to participate in the trial by completing the online screening measures and 13 (43.3%) were eligible, consented to participate and were enrolled in the trial.

#### 3.2.2. Trial Attrition

All 13 participants enrolled in the trial completed the pre-treatment questionnaires. Nine participants (69.2%) completed all of the post-treatment questionnaires. An additional participant completed a subset of the post-treatment questionnaires (specifically, measures of mental health and RTW outcomes, but did not complete measures of satisfaction or usability). Thus, four participants (30.8%) did not complete all of the post-treatment questionnaires.

#### 3.2.3. Program Adherence

A total of 12 out of 13 participants (92.3%) completed all six core online modules of the *Workable* program. One participant dropped out after the second week (after completing two modules and participating in two coaching calls) and did not provide a reason for doing so or complete post-treatment questionnaires. Seven participants (53.8%) also completed at least one of the optional *Workable* modules related to pain and fatigue management.

The 12 participants who completed the 6 core *Workable* modules also participated in all 6 coaching calls with an Uprise psychologist. All coaching calls were conducted via videoconference, with the exception of one participant who received coaching calls via landline telephone, due to poor Internet and mobile phone reception.

#### 3.2.4. Safety Protocol

At pre-treatment, the safety protocol was triggered by 11 participants (84.6%) scoring under 8/25 on the WHO-5 measure of well-being. These participants received a check-in call from a psychologist. The safety protocol was also triggered at post-treatment, with three participants (23.0%) scoring below the cut-off of 8/25 on the WHO-5 and receiving a check-in call. No participants reported current suicidal ideation to the trial psychologist at any stage of the study.

### 3.3. Acceptability

#### 3.3.1. Satisfaction (n = 9)

The mean CSQ-8 score was 26.89 (SD = 3.02, range = 23–32). According to a previously reported classification of CSQ-8 scores [33], this is in the “satisfied” range. All 9 participants who completed the CSQ-8 reported that they would recommend *Workable* to a friend in need of similar help. Additionally, all reported that they were either ‘mostly’ (55.6%) or ‘very’ (44.4%) satisfied overall with the program. All respondents also reported that *Workable* helped them to deal with their problems more effectively, either ‘a great deal’ (33.3%) or ‘somewhat’ (66.7%).

#### 3.3.2. Usability (n = 9)

All participants who completed the post-treatment questionnaires indicated that they either ‘strongly agreed’ (33.3%) or ‘agreed’ (66.7%) that *Workable* was very easy to use.

#### 3.3.3. Qualitative Online Feedback

##### Most Helpful Parts of Workable (n = 9)

Responses regarding the most helpful aspects of the *Workable* program fell into three main categories: (i) both online content and the coaching calls with a psychologist (44.4%); (ii) specifically the coaching calls with a psychologist (22.2%); (iii) specifically the videos in the online modules. One participant did not respond accurately to the question (11.1%), An example of the feedback is as follows:

*The most helpful was the information disseminated and the exercises that followed. They were short and effective. It was not long or complicated so easy to follow and made me think about the exercises which were helpful*.

##### Least Helpful Parts of Workable (n = 9)

Regarding the least helpful aspects of the program, three participants (33.3%) reported a technical issue with *Workable* incorrectly informing them that they had more work to complete when they had in fact completed it, for example:

*The system could update the programs better. It continuously said that I had tasks to finish but everything was completed. So that was the only thing that I found confusing and did not like.*

One (11.1%) reported that the system was “a bit clunky” and another (11.1%) that the questions in exercises were “too automatic”. Two participants (22.2%) reported that they had difficulties with concentration, whilst another two (22.2%) reported that there were no aspects of *Workable* they found least helpful:

*Nothing was least helpful. I was not discouraged at any time during the course.*

##### Suggestions for Improvement (n = 8)

Four participants (50.0%) reported that they did not have any suggestions for improvement. Two (25.0%) reported that technical issues in the questions within program exercises should be addressed, and another participant (12.5%) suggested that technical issues should be fixed (but did not specify the issues). Finally, one participant (12.5%) made several suggestions for improvement, including offering the program face-to-face instead of online, adding more exercises, and adding more options to provide comments:

*To me it is an excellent program but to others it may be intimidating. If it is conducted face-to-face it may have more impact and give satisfaction to a receiver.*

##### General Comments (n = 4)

Of those who provided further comments, two participants (50.0%) expressed thanks to the psychologist who provided them with coaching calls throughout the *Workable* program. Two participants (50.0%) provided positive comments, with one also mentioning that *Workable* was easy to use despite minor technical issues:

*Thank you for your support. I was aware of CBT but your program gave me more skills and activities to follow and they have been helpful. I forgot some of the steps before but this program showed me ways to go back and do things that I did not think about. Overall I benefited from the program and will follow the activities suggested.*

### 3.4. Potential Effectiveness: Mental Health Outcomes

Table 4 reports the results of linear mixed model analyses for each of the secondary outcome measures at pre- and post-treatment. The improvements in symptoms of depression, anxiety, and stress from pre- to post-treatment were all statistically significant (*p* < 0.05), with large effect sizes. The reduction in symptoms of depression from pre- to post-treatment (assessed by scores on the PHQ-9) was statistically significant (*p* < 0.05), with a large effect size (Hedges *g* = 1.16). The reductions in symptoms of anxiety and stress from pre- to post-treatment (assessed by scores on the GAD-7 and PSS-4, respectively) were also statistically significant (*p* < 0.05), with large effect sizes (Hedges *g* = 1.10 and 1.32, respectively). There was a non-significant trend (*p* = 0.064) towards an increase in scores on the WHO-5 (with higher scores indicating better well-being) from pre- to post-treatment, with a large effect size (Hedges *g* = 0.84).

### 3.5. Potential Effectiveness: RTW Outcomes

Table 4 reports the results of linear mixed model analyses for work ability and work absence at pre- and post-treatment. The increase in self-reported current work ability from pre- to post-treatment was statistically significant (*p* < 0.05), with a moderately large effect size (Hedges *g* = 0.73). The decrease from pre- to post-treatment in the mean number of days absent from work over the past four weeks, due to symptoms, was statistically significant at the *p* < 0.01 level, with a large effect size (Hedges *g* = 1.69).

Ten participants completed the post-treatment questions regarding their current working status. Of these 10 participants, 60.0% reported at post-treatment (6 weeks after commencing the *Workable* program), that they were back at work. Amongst these, one participant was working full-time, three part-time, and two casually. Five participants reported that they were working for the same employer as prior to their sick leave, and one participant was working for a new employer.

## 4. Discussion

This pilot trial aimed to explore the feasibility and acceptability of *Workable*, a 6-week digital program with therapist support for injured workers. In addition, a secondary aim was to conduct a preliminary examination of the impact of the program on mental health and RTW outcomes at post-treatment (6 weeks).

The findings from this pilot trial suggest that *Workable* is a feasible and acceptable intervention for workers from a broad range of industries on sick leave for a variety of psychological or physical complaints. The results also suggest that the processes for recruitment and gathering data from participants are feasible, leading to a moderate recruitment rate. However, it can be noted that 40% of the injured workers informed of the pilot trial of *Workable* chose not to apply. Amongst participants who did enroll in the trial, the program adherence rate was very high, with 92% of participants completing the 6-week *Workable* program and participating in all six coaching calls with a psychologist. Further, participants reported being satisfied with *Workable* and finding it easy to use. Qualitative feedback from participants was consistent with the quantitative ratings of satisfaction. Both face-to-face and online aspects of the program were well-received. Several participants specifically stated that they found the content of *Workable* (videos and exercises) helpful and easy to understand. Whilst two participants stated that they had problems with concentration, they still completed all 6 modules and participated in all 6 coaching calls. Minor technical difficulties were the main program criticisms raised.

A secondary aim of this pilot trial was to conduct a preliminary investigation of the impact of the 6-week *Workable* program on mental health and RTW outcomes. There were large and significant reductions between pre-treatment and post-treatment on measures of depression, anxiety, and stress. There was also a large but statistically insignificant increase on a measure of well-being, and it is possible that this increase in well-being may have reached statistical significance with a larger sample size. The potential effectiveness of the *Workable* program in reducing depression, anxiety, and stress is important not only for the mental health benefits in their own right, but also given that symptoms of CMD are known to significantly reduce the likelihood of returning to work [9,14].

Results were also promising for RTW outcomes. Participating in the *Workable* program was followed by a moderately large and significant increase in self-reported work ability, and a large and significant decrease in the mean number of full working days missed in the past four weeks. Notably, 60% of post-questionnaire respondents reported that they were back at work by the end of the 6-week *Workable* program. Although these results should be treated with caution given the lack of control group and other limitations as described below, they are in line with previous findings that psychological interventions with a specific work focus can be effective in improving RTW outcomes [19,20,24].

It is not known which elements of the *Workable* program are most important for both engagement and any benefits. For example, it is possible that there would have had the same degree of acceptability and possible benefits for participants without the weekly coaching calls provided by a psychologist. Similarly, it is possible that the weekly coaching calls were in fact the most important component. However, there is substantial evidence that therapist-supported online CBT is more effective than unguided CBT for reducing depression [50]. Further, the qualitative feedback from some participants emphasized the benefits of the weekly coaching calls from a psychologist, which suggests that they may be an important component of the blended program.

There are several limitations of this trial to be considered. Firstly, the uncontrolled nature of the trial limits conclusions around efficacy. Such pilot studies are a fundamental phase of clinical research and play a key role in the refinement of new interventions and informing the design of future RCTs to examine efficacy [51]. Whilst a preliminary examination of the impact of *Workable* on mental health and RTW outcomes was included in this pilot trial, it should be noted that the results cannot demonstrate causality and it is not known if the benefits of *Workable* would be found compared to a control group.

A further limitation of this pilot trial was that the assessment of outcomes relied on self-report data. In order to minimize the burden on participants from completing questionnaires during this pilot trial, only a subset of items from the Work Ability Index (WAI) was administered. Future investigations of the efficacy of *Workable* could include the full set of items from the WAI and/or other RTW outcome measures, given that the full WAI has been found to have greater ability to predict long term sickness absence than its individual items [49].

Further, the sample size and eligibility criteria may limit the generalizability of the results. Whilst the sample size was adequate for assessing feasibility and acceptability, and in line with other feasibility pilot trials of online interventions [26,27,28,29,30], it consisted of 9 participants on sick leave for a psychological injury and 4 participants on leave for a physical injury. Thus, there was not a sufficient sample size to also conduct sub-analyses for those two groups. It is not known if the results would have differed if the sample had only included those on leave for a mental health issue, or only those on leave for a physical injury. Further, this study excluded participants with severe depression or suicidal intent or plan, and none of the participants expressed suicidal ideation to the trial psychologist. The mean baseline scores of participants on measures of depression (PHQ-9) and anxiety (GAD-7) were in the moderate range. Therefore, it is not known if *Workable* would meet the needs of injured workers with more severe mental health difficulties. Moreover, participants were injured workers with a claim duration of 2 to 26 weeks at the time of recruitment, and over two thirds were on leave for a psychological injury. It is not known what the impacts of *Workable* would be among injured workers with a claim duration of greater than 26 weeks. Given that longer absences from work are associated with a reduced probability of eventual return [4], it may be that the RTW outcomes following *Workable* are different in those with a longer claim duration.

Future research should include an RCT to examine the effects of *Workable* on mental health and RTW outcomes compared to care as usual (CAU), using validated RTW measures and a longer-term follow-up. An evaluation of cost-effectiveness should be included in such an RCT. This kind of larger study could examine predictors of response to *Workable,* such as the duration of sick leave and whether leave was for a physical or psychological injury. Such trials could also look to investigate the relative importance of therapist and digital components of this blended model of care. However, prior to an investigation of the efficacy of *Workable*, the minor technical difficulties identified in this study should be addressed. Finally, future investigations may consider examining the perspectives of injured workers who choose not to apply to take part in *Workable* to examine any barriers to participation and how their needs could be met.

## 5. Conclusions

The results from this pilot trial suggest that *Workable,* a therapist-assisted Web-based program, is a feasible and acceptable intervention for injured workers on sick leave for a psychological or physical injury. Preliminary results of this pilot study suggest that *Workable* may be helpful for reducing depression, anxiety, and stress in injured workers and may have the potential to improve RTW outcomes. An RCT is required to determine the efficacy of the *Workable* program.

## Figures and Tables

**Table 1:** Content of the Web-based *Workable* program.

Week	Module	Content
**Core Modules**		
Weeks 1 and 2	Mindset	Introduction to cognitive behavioural therapy (CBT) including the ABC model and principles of cognitive restructuring.
	Stress Management	Application of CBT skill of cognitive restructuring to thoughts that cause stress, including thoughts related to returning to work or problems at work (e.g., thoughts about a colleague or manager being unsupportive or concerns about duties or pain management).
	Worry Time	Instructions on how to use the skill of ‘worry time’ or actively writing about worrying thoughts and ignoring at other times.
Weeks 3 and 4	Overview	Introduction and summary of problem solving therapy (PST).
	Problem Definition	Instructions on how to define a problem using PST, including application to perceived barriers to returning to work.
	Solution Selection	Instructions on how to rank solutions into categories based on viability of the solution, including application to possible solutions to perceived barriers to returning to work.
	Experimentation	Instructions on how to collect evidence about solutions to restructure maladaptive efficacy beliefs.
Weeks 5 and 6	Smart Goals	Summary of the SMART goals approach.
	Planning for Success	Instructions on how to manage relapses using the skills from CBT.
** Optional Modules **		
At any time	Pain Overview	Summary of pacing and graded exposure skills.
	Pacing	Instructions on how to pace activities to prevent inactivity due to overactivity.
	Stepladders	Instructions on using graded exposure.
	Sleep Basics	Instructions on sleep hygiene.

**Table 2:** Schedule of assessment measures.

Measures	Pre	Post
Demographic variables	✕	
**Acceptability**		
CSQ-8 (Satisfaction)		✕
Usability item		✕
Qualitative online feedback		✕
**Feasibility**		
Recruitment rate	✕	
Trial attrition rate		✕
Adherence: Number of online modules		✕
Adherence: Number of coaching calls		✕
**Mental Health Outcomes**		
PHQ-9 (Depression)	✕	✕
GAD-7 (Anxiety)	✕	✕
PSS-4 (Stress)	✕	✕
WHO-5 (Well-being)	✕	✕
**RTW Outcomes**		
Work ability	✕	✕
Absence from work	✕	✕
RTW status		✕

Note: CSQ-8 = Client Satisfaction Questionnaire; PHQ-9 = Patient Health Questionnaire-9; GAD-7 = Generalised Anxiety Disorder-7; PSS-4 = Perceived Stress Scale-4; WHO-5 = WHO-5 Well-being Index; RTW = Return-to-Work; Work ability = 3 items from the WAI (Work Ability Index); Absence from work = Number of full working days in the past 4 weeks that have been missed due to symptoms.

**Table 3:** Baseline participant characteristics.

Baseline Characteristic	Total Sample (N = 13)
Age, mean (SD)	47.54 (13.43)
Gender, n (%)	
Female	6 (46.2)
Male	7 (53.8)
Education, n (%)	
Some high school, no certificate	2 (15.4)
High school graduate, diploma or equivalent	6 (46.2)
Some university, no degree	2 (15.4)
Trade, technical or vocational training	1 (7.7)
Bachelor degree	2 (15.4)
Reason for sick leave, n (%)	
Psychological injury	9 (69.2)
Joint/ligament or muscle/tendon injury	2 (15.4)
Infectious or parasitic disease	1 (7.7)
Spinal cord or nerve injury	1 (7.7)
PHQ-9 (depression), mean (SD)	14.00 (4.16)
GAD-7 (anxiety), mean (SD)	12.15 (3.91)

Note: PHQ-9 = Patient Health Questionnaire-9; GAD-7 = Generalized Anxiety Disorder-7.

**Table 4:** Estimated marginal means at pre-treatment and post-treatment following *Workable*.

	Pre-Treatment		Post-Treatment		Pre- to Post- Treatment			
							Effect Size	
	n	Mean (SD)	n	Mean (SD)	*F* (df)	*p*	Hedges *g*	95% CI
Mental health								
Depression (PHQ-9)	13	14.00 (4.16)	10	8.09 (5.7)	10.39 (1, 9.63)	0.010	1.16	0.29–2.09
Anxiety (GAD-7)	13	12.15 (3.64)	10	6.90 (5.63)	8.81 (1, 8.49)	0.017	1.10	0.24–2.02
Stress (PSS-4)	13	10.02 (1.71)	10	6.90 (5.63)	10.61 (1, 9.68)	0.009	1.32	0.44–2.28
Well-being (WHO-5)	13	7.00 (3.19)	10	11.00 (6.01)	4.39 (1, 9.44)	0.064	0.84	−0.01–1.72
RTW								
Work ability	13	9.46 (3.69)	10	12.45 (4.23)	7.35 (1, 8.21)	0.026	0.73	−0.10–1.61
Absence from work	13	25.54 (6.06)	10	11.16 (10.42)	22.49 (1, 8.90)	0.001	1.69	0.76–2.71

Note: PHQ-9 = Patient Health Questionnaire-9; GAD-7 = Generalized Anxiety Disorder-7; PSS-4 = Perceived Stress Scale-4; WHO-5 = WHO-5 Well-being Index; RTW = Return-to-Work; Work ability = 3 items from the WAI (Work Ability Index); Absence from work = Number of full working days in the past 4 weeks that have been missed due to symptoms.

## Data Availability

The data are not publicly available due to ethical restrictions.
